# THE INFLUENCE OF TECHNOLOGY ON QUALITY OF LIFE AND AGING IN PLACE

**DOI:** 10.1093/geroni/igz038.1201

**Published:** 2019-11-08

**Authors:** George Mois, Tiffany R Washington, Jenay M Beer

**Affiliations:** University of Georgia, Athens, Georgia, United States

## Abstract

Many of the challenges that often accompany longevity can affect older adults’ quality of life (QOL). Adoption of an assistive technology ecosystem presents the potential to alleviate these challenges and improve QOL. An assistive technology ecosystem refers to the use of multiple assistive technologies to address a set of challenges affecting single or multiple characteristics of older adults’ QOL. However, little is known how technology can influence characteristics of older adult’s QOL. The purpose of this study was to investigate how using technology can improve older adults QOL. Data from the 2016 wave of the National Health and Aging Trends Study (NHATS) were analyzed using four logistic regression models. The sample included are older adults age 65+ (N=5,488). The dependent variables used in this study were QOL indicators such as self-confidence, continue improving life, likes living arrangement, and self-determination. The variables used to measure technology included computer, cell phone, tablet, and internet use. Older adults who used the internet had significantly higher odds of reporting self-determination (OR=1.68), like living arrangement (OR= 1.97) and continue improving life. Tablet users had significantly higher odds of continuing to improve their life (OR= 1.249) and increased self-determination (OR= 1.174). Cellphone users had significantly higher odds of having self-confidence (OR= 2.814). These findings support the need for a network of resources accessed through an ecosystem of technologies to address the challenges encountered by older adults aging in place. This study’s findings can inform technology education programs, interventions, and assist with the development of support networks.

